# Bis[4-(dimethyl­amino)pyridinium] tetra­bromidocadmate(II) monohydrate

**DOI:** 10.1107/S1600536809014366

**Published:** 2009-04-25

**Authors:** Kong Mun Lo, Seik Weng Ng

**Affiliations:** a Department of Chemistry, University of Malaya, 50603 Kuala Lumpur, Malaysia

## Abstract

The Cd atom in the hydrated title salt, (C_7_H_11_N_2_)_2_[CdBr_4_]·H_2_O, exists in an approximately tetra­hedral coordination geometry, with Br—Cd—Br angles in the range 105.52 (3)–111.50 (3)°. The cation, anion and water mol­ecule inter­act by O—H⋯Br, N—H⋯Br and N—H⋯O hydrogen bonds, forming a linear chain structure running along the *a* axis.

## Related literature

For other tetra­hedral ammonium tetra­bromidocadmates, see: Altermatt *et al.* (1979 ▶); Battaglia *et al.* (1991 ▶); Casals *et al.* (1987 ▶); Geselle & Fuess (1994 ▶); Ishihara *et al.* (1998 ▶); Krishnan *et al.* (1991 ▶); Sato *et al.* (1986 ▶); Waskowska (1994 ▶).
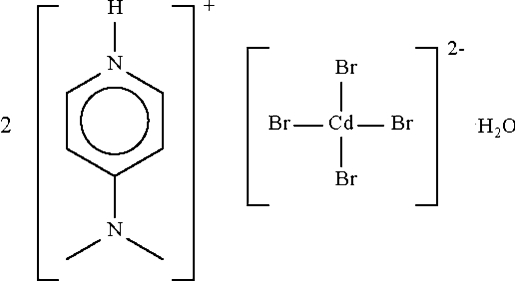


## Experimental

### 

#### Crystal data

(C_7_H_11_N_2_)_2_[CdBr_4_]·H_2_O
*M* *_r_* = 696.41
Triclinic, 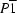

*a* = 7.8918 (2) Å
*b* = 8.1197 (2) Å
*c* = 17.2719 (4) Å
α = 95.481 (1)°
β = 99.747 (1)°
γ = 96.489 (1)°
*V* = 1076.35 (5) Å^3^
*Z* = 2
Mo *K*α radiation
μ = 8.45 mm^−1^
*T* = 100 K
0.25 × 0.25 × 0.25 mm

#### Data collection

Bruker SMART APEX diffractometer
Absorption correction: multi-scan (*SADABS*; Sheldrick, 1996 ▶) *T*_min_ = 0.258, *T*_max_ = 0.431 (expected range = 0.072–0.121)
6014 measured reflections
3711 independent reflections
3255 reflections with *I* > 2σ(*I*)
*R*_int_ = 0.022

#### Refinement

*R*[*F* ^2^ > 2σ(*F* ^2^)] = 0.037
*wR*(*F* ^2^) = 0.131
*S* = 1.07
3711 reflections
221 parameters
H-atom parameters constrained
Δρ_max_ = 1.53 e Å^−3^
Δρ_min_ = −1.65 e Å^−3^

### 

Data collection: *APEX2* (Bruker, 2008 ▶); cell refinement: *APEX2*; data reduction: *SAINT* (Bruker, 2008 ▶); program(s) used to solve structure: *SHELXS97* (Sheldrick, 2008 ▶); program(s) used to refine structure: *SHELXL97* (Sheldrick, 2008 ▶); molecular graphics: *X-SEED* (Barbour, 2001 ▶); software used to prepare material for publication: *pubCIF* (Westrip, 2009 ▶).

## Supplementary Material

Crystal structure: contains datablocks global, I. DOI: 10.1107/S1600536809014366/sj2619sup1.cif

Structure factors: contains datablocks I. DOI: 10.1107/S1600536809014366/sj2619Isup2.hkl

Additional supplementary materials:  crystallographic information; 3D view; checkCIF report

## Figures and Tables

**Table 1:** Selected bond angles (°)

Br1—Cd1—Br2	109.23 (3)
Br1—Cd1—Br3	109.83 (3)
Br4—Cd1—Br1	111.50 (3)
Br2—Cd1—Br3	105.52 (3)
Br2—Cd1—Br4	110.72 (3)
Br3—Cd1—Br4	109.87 (3)

**Table 2:** Hydrogen-bond geometry (Å, °)

*D*—H⋯*A*	*D*—H	H⋯*A*	*D*⋯*A*	*D*—H⋯*A*
O1—H11⋯Br1	0.84	2.83	3.351 (7)	122
O1—H12⋯Br3^i^	0.84	2.67	3.511 (7)	172
N1—H1⋯Br2	0.88	2.56	3.373 (7)	154
N3—H3⋯O1	0.88	2.36	3.011 (10)	131

Symmetry code: (i) 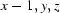.

**Fig. 1.**
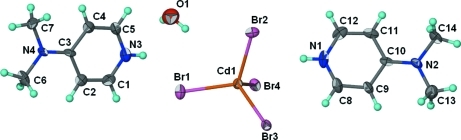
Thermal ellipsoid plot (Barbour, 2001) of (C7H11N2)2(CdBr4).H2O at the 70% probability level. H atoms are drawn as spheres of arbitrary radius.

## supplementary crystallographic information

### Experimental

Cadmium chloride (0.5 g, 2 mmol) dissolved in water (10 ml) and 4-dimethylaminopyridine hydrobromide perbromide (0.8 g, 2 mmol) dissolved in ethanol (80 ml) were mixed and the mixture heated for 1 h. Slow evaporation of the filtrate gave colorless crystals.

### Refinement

Carbon- and nitrogen-bound H atoms were placed in calculated positions (C—H 0.95 to 0.98 Å, N—H 0.88 Å) and were included in the refinement in the riding model approximation, with *U*(H) fixed at 1.2–1.5*U*(C). The water H atoms were placed in chemically sensible positions on the basis of hydrogen-bonding interactions, but were not refined.

The final difference Fourier map had a large peak at 1.3 Å from H5 and a deep hole at 0.7 Å from Br1.

### Crystal data

**Table:**

(C_7_H_11_N_2_)_2_[CdBr_4_]·H_2_O	*Z* = 2
*M**_r_* = 696.41	*F*(000) = 664
Triclinic, *P*1	*D*_x_ = 2.149 Mg m^−^^3^
Hall symbol: -P 1	Mo *K*α radiation, λ = 0.71073 Å
*a* = 7.8918 (2) Å	Cell parameters from 3763 reflections
*b* = 8.1197 (2) Å	θ = 2.4–28.2°
*c* = 17.2719 (4) Å	µ = 8.45 mm^−^^1^
α = 95.481 (1)°	*T* = 100 K
β = 99.747 (1)°	Irregular block, colourless
γ = 96.489 (1)°	0.25 × 0.25 × 0.25 mm
*V* = 1076.35 (5) Å^3^	

### Data collection

**Table:**

Bruker SMART APEX diffractometer	3711 independent reflections
Radiation source: fine-focus sealed tube	3255 reflections with *I* > 2σ(*I*)
graphite	*R*_int_ = 0.022
ω scans	θ_max_ = 25.0°, θ_min_ = 2.4°
Absorption correction: multi-scan (*SADABS*; Sheldrick, 1996)	*h* = −9→9
*T*_min_ = 0.258, *T*_max_ = 0.431	*k* = −9→9
6014 measured reflections	*l* = −20→20

### Refinement

**Table:**

Refinement on *F*^2^	Primary atom site location: structure-invariant direct methods
Least-squares matrix: full	Secondary atom site location: difference Fourier map
*R*[*F*^2^ > 2σ(*F*^2^)] = 0.037	Hydrogen site location: inferred from neighbouring sites
*wR*(*F*^2^) = 0.131	H-atom parameters constrained
*S* = 1.07	*w* = 1/[σ^2^(*F*_o_^2^) + (0.0774*P*)^2^ + 6.748*P*] where *P* = (*F*_o_^2^ + 2*F*_c_^2^)/3
3711 reflections	(Δ/σ)_max_ = 0.001
221 parameters	Δρ_max_ = 1.53 e Å^−^^3^
0 restraints	Δρ_min_ = −1.65 e Å^−^^3^

### Fractional atomic coordinates and isotropic or equivalent isotropic displacement parameters (Å^2^)

**Table:**

	*x*	*y*	*z*	*U*_iso_*/*U*_eq_	
Cd1	0.56389 (6)	0.31451 (6)	0.25658 (3)	0.01616 (17)	
Br1	0.43625 (10)	0.44170 (10)	0.37244 (5)	0.0256 (2)	
Br2	0.53082 (10)	0.50457 (9)	0.14316 (5)	0.0237 (2)	
Br3	0.89498 (9)	0.31530 (10)	0.30001 (4)	0.0205 (2)	
Br4	0.41821 (9)	0.01732 (9)	0.20678 (4)	0.0209 (2)	
O1	0.1769 (9)	0.6316 (8)	0.2453 (4)	0.0438 (17)	
H11	0.2666	0.5854	0.2431	0.066*	
H12	0.1005	0.5627	0.2575	0.066*	
N1	0.8619 (9)	0.3422 (9)	0.0770 (4)	0.0280 (16)	
H1	0.7974	0.3812	0.1095	0.034*	
N2	1.1657 (8)	0.1572 (8)	−0.0724 (4)	0.0187 (13)	
N3	0.2927 (8)	0.8098 (8)	0.4100 (4)	0.0233 (14)	
H3	0.3098	0.7253	0.3777	0.028*	
N4	0.1981 (8)	1.1958 (7)	0.5626 (3)	0.0150 (12)	
C1	0.3353 (10)	0.8102 (9)	0.4877 (5)	0.0210 (16)	
H1A	0.3895	0.7197	0.5070	0.025*	
C2	0.3061 (9)	0.9320 (9)	0.5412 (5)	0.0199 (16)	
H2	0.3352	0.9246	0.5963	0.024*	
C3	0.2300 (9)	1.0730 (9)	0.5130 (4)	0.0143 (14)	
C4	0.1916 (9)	1.0680 (9)	0.4308 (5)	0.0179 (15)	
H4	0.1414	1.1579	0.4089	0.022*	
C5	0.2228 (9)	0.9411 (9)	0.3813 (4)	0.0197 (15)	
H5	0.1955	0.9438	0.3258	0.024*	
C6	0.2363 (11)	1.1943 (10)	0.6469 (5)	0.0258 (18)	
H6A	0.1801	1.0901	0.6608	0.039*	
H6B	0.1928	1.2893	0.6729	0.039*	
H6C	0.3620	1.2025	0.6645	0.039*	
C7	0.1264 (10)	1.3411 (9)	0.5317 (5)	0.0195 (15)	
H7A	0.2057	1.3956	0.5012	0.029*	
H7B	0.1122	1.4203	0.5759	0.029*	
H7C	0.0134	1.3043	0.4975	0.029*	
C8	1.0058 (10)	0.2831 (10)	0.1052 (5)	0.0249 (17)	
H8	1.0381	0.2845	0.1608	0.030*	
C9	1.1099 (10)	0.2201 (10)	0.0575 (4)	0.0199 (15)	
H9	1.2124	0.1779	0.0800	0.024*	
C10	1.0651 (9)	0.2175 (8)	−0.0254 (4)	0.0163 (15)	
C11	0.9084 (10)	0.2811 (9)	−0.0549 (5)	0.0221 (17)	
H11A	0.8709	0.2804	−0.1102	0.026*	
C12	0.8130 (10)	0.3430 (9)	−0.0026 (5)	0.0245 (17)	
H12A	0.7100	0.3877	−0.0220	0.029*	
C13	1.3311 (10)	0.1038 (11)	−0.0401 (5)	0.0274 (18)	
H13A	1.3938	0.1870	0.0031	0.041*	
H13B	1.4006	0.0925	−0.0818	0.041*	
H13C	1.3100	−0.0040	−0.0199	0.041*	
C14	1.1133 (11)	0.1373 (10)	−0.1580 (4)	0.0242 (17)	
H14A	1.1968	0.0788	−0.1822	0.036*	
H14B	1.1100	0.2474	−0.1766	0.036*	
H14C	0.9979	0.0723	−0.1728	0.036*	

### Atomic displacement parameters (Å^2^)

**Table:**

	*U*^11^	*U*^22^	*U*^33^	*U*^12^	*U*^13^	*U*^23^
Cd1	0.0151 (3)	0.0170 (3)	0.0153 (3)	0.0015 (2)	0.0019 (2)	−0.0014 (2)
Br1	0.0261 (4)	0.0246 (4)	0.0270 (5)	0.0027 (3)	0.0108 (3)	−0.0031 (3)
Br2	0.0229 (4)	0.0236 (4)	0.0244 (4)	0.0049 (3)	0.0000 (3)	0.0067 (3)
Br3	0.0158 (4)	0.0304 (4)	0.0148 (4)	0.0038 (3)	0.0021 (3)	0.0002 (3)
Br4	0.0239 (4)	0.0184 (4)	0.0187 (4)	−0.0008 (3)	0.0038 (3)	−0.0013 (3)
O1	0.049 (4)	0.037 (4)	0.044 (4)	0.010 (3)	0.004 (3)	0.000 (3)
N1	0.025 (4)	0.027 (4)	0.034 (4)	−0.001 (3)	0.019 (3)	−0.005 (3)
N2	0.020 (3)	0.026 (3)	0.011 (3)	0.002 (3)	0.004 (2)	0.007 (3)
N3	0.024 (3)	0.024 (3)	0.023 (4)	0.000 (3)	0.010 (3)	0.000 (3)
N4	0.019 (3)	0.017 (3)	0.009 (3)	0.003 (2)	0.001 (2)	0.004 (2)
C1	0.022 (4)	0.016 (4)	0.028 (4)	0.004 (3)	0.007 (3)	0.013 (3)
C2	0.016 (3)	0.019 (4)	0.024 (4)	−0.002 (3)	0.003 (3)	0.003 (3)
C3	0.012 (3)	0.017 (3)	0.013 (4)	−0.002 (3)	−0.001 (3)	0.012 (3)
C4	0.011 (3)	0.020 (4)	0.022 (4)	−0.001 (3)	0.002 (3)	0.003 (3)
C5	0.021 (4)	0.023 (4)	0.015 (4)	0.000 (3)	0.007 (3)	0.001 (3)
C6	0.031 (4)	0.027 (4)	0.017 (4)	−0.008 (3)	0.010 (3)	−0.006 (3)
C7	0.023 (4)	0.013 (3)	0.023 (4)	0.006 (3)	0.003 (3)	−0.001 (3)
C8	0.026 (4)	0.026 (4)	0.022 (4)	−0.002 (3)	0.009 (3)	−0.007 (3)
C9	0.022 (4)	0.025 (4)	0.012 (4)	0.000 (3)	0.005 (3)	−0.001 (3)
C10	0.019 (4)	0.011 (3)	0.016 (4)	−0.006 (3)	−0.004 (3)	0.008 (3)
C11	0.020 (4)	0.021 (4)	0.020 (4)	−0.010 (3)	−0.005 (3)	0.009 (3)
C12	0.020 (4)	0.015 (4)	0.037 (5)	0.000 (3)	0.003 (3)	0.005 (3)
C13	0.022 (4)	0.028 (4)	0.031 (5)	0.002 (3)	0.003 (3)	0.001 (4)
C14	0.031 (4)	0.028 (4)	0.012 (4)	0.001 (3)	0.003 (3)	0.001 (3)

### Geometric parameters (Å, °)

**Table:**

Cd1—Br1	2.5706 (9)	C4—C5	1.347 (11)
Cd1—Br2	2.6044 (9)	C4—H4	0.9500
Cd1—Br3	2.5926 (8)	C5—H5	0.9500
Cd1—Br4	2.5546 (8)	C6—H6A	0.9800
O1—H11	0.8418	C6—H6B	0.9800
O1—H12	0.8427	C6—H6C	0.9800
N1—C8	1.318 (11)	C7—H7A	0.9800
N1—C12	1.364 (11)	C7—H7B	0.9800
N1—H1	0.8800	C7—H7C	0.9800
N2—C10	1.326 (10)	C8—C9	1.364 (11)
N2—C14	1.455 (10)	C8—H8	0.9500
N2—C13	1.460 (10)	C9—C10	1.414 (10)
N3—C1	1.327 (10)	C9—H9	0.9500
N3—C5	1.352 (10)	C10—C11	1.427 (11)
N3—H3	0.8800	C11—C12	1.367 (12)
N4—C3	1.325 (10)	C11—H11A	0.9500
N4—C6	1.438 (10)	C12—H12A	0.9500
N4—C7	1.472 (9)	C13—H13A	0.9800
C1—C2	1.355 (11)	C13—H13B	0.9800
C1—H1A	0.9500	C13—H13C	0.9800
C2—C3	1.439 (10)	C14—H14A	0.9800
C2—H2	0.9500	C14—H14B	0.9800
C3—C4	1.396 (10)	C14—H14C	0.9800
			
Br1—Cd1—Br2	109.23 (3)	N4—C6—H6C	109.5
Br1—Cd1—Br3	109.83 (3)	H6A—C6—H6C	109.5
Br4—Cd1—Br1	111.50 (3)	H6B—C6—H6C	109.5
Br2—Cd1—Br3	105.52 (3)	N4—C7—H7A	109.5
Br2—Cd1—Br4	110.72 (3)	N4—C7—H7B	109.5
Br3—Cd1—Br4	109.87 (3)	H7A—C7—H7B	109.5
H11—O1—H12	108.9	N4—C7—H7C	109.5
C8—N1—C12	120.1 (7)	H7A—C7—H7C	109.5
C8—N1—H1	120.0	H7B—C7—H7C	109.5
C12—N1—H1	120.0	N1—C8—C9	122.4 (8)
C10—N2—C14	121.3 (6)	N1—C8—H8	118.8
C10—N2—C13	121.3 (6)	C9—C8—H8	118.8
C14—N2—C13	117.4 (6)	C8—C9—C10	119.8 (7)
C1—N3—C5	119.1 (7)	C8—C9—H9	120.1
C1—N3—H3	120.5	C10—C9—H9	120.1
C5—N3—H3	120.5	N2—C10—C9	120.4 (7)
C3—N4—C6	121.8 (6)	N2—C10—C11	122.7 (7)
C3—N4—C7	120.0 (6)	C9—C10—C11	116.9 (7)
C6—N4—C7	118.2 (6)	C12—C11—C10	119.1 (7)
N3—C1—C2	123.8 (7)	C12—C11—H11A	120.4
N3—C1—H1A	118.1	C10—C11—H11A	120.4
C2—C1—H1A	118.1	N1—C12—C11	121.6 (7)
C1—C2—C3	118.8 (7)	N1—C12—H12A	119.2
C1—C2—H2	120.6	C11—C12—H12A	119.2
C3—C2—H2	120.6	N2—C13—H13A	109.5
N4—C3—C4	123.6 (6)	N2—C13—H13B	109.5
N4—C3—C2	121.4 (6)	H13A—C13—H13B	109.5
C4—C3—C2	114.9 (7)	N2—C13—H13C	109.5
C5—C4—C3	122.9 (7)	H13A—C13—H13C	109.5
C5—C4—H4	118.6	H13B—C13—H13C	109.5
C3—C4—H4	118.6	N2—C14—H14A	109.5
C4—C5—N3	120.5 (7)	N2—C14—H14B	109.5
C4—C5—H5	119.8	H14A—C14—H14B	109.5
N3—C5—H5	119.8	N2—C14—H14C	109.5
N4—C6—H6A	109.5	H14A—C14—H14C	109.5
N4—C6—H6B	109.5	H14B—C14—H14C	109.5
H6A—C6—H6B	109.5		
			
C5—N3—C1—C2	3.0 (11)	C12—N1—C8—C9	−0.5 (12)
N3—C1—C2—C3	−2.3 (11)	N1—C8—C9—C10	0.4 (12)
C6—N4—C3—C4	178.6 (7)	C14—N2—C10—C9	173.5 (7)
C7—N4—C3—C4	−3.1 (10)	C13—N2—C10—C9	−4.7 (10)
C6—N4—C3—C2	−0.5 (10)	C14—N2—C10—C11	−6.1 (10)
C7—N4—C3—C2	177.7 (6)	C13—N2—C10—C11	175.7 (7)
C1—C2—C3—N4	179.9 (7)	C8—C9—C10—N2	179.7 (7)
C1—C2—C3—C4	0.6 (10)	C8—C9—C10—C11	−0.7 (10)
N4—C3—C4—C5	−179.1 (7)	N2—C10—C11—C12	−179.2 (7)
C2—C3—C4—C5	0.1 (10)	C9—C10—C11—C12	1.2 (10)
C3—C4—C5—N3	0.6 (11)	C8—N1—C12—C11	1.1 (11)
C1—N3—C5—C4	−2.1 (11)	C10—C11—C12—N1	−1.4 (11)

### Hydrogen-bond geometry (Å, °)

**Table:**

*D*—H···*A*	*D*—H	H···*A*	*D*···*A*	*D*—H···*A*
O1—H11···Br1	0.84	2.83	3.351 (7)	122
O1—H12···Br3^i^	0.84	2.67	3.511 (7)	172
N1—H1···Br2	0.88	2.56	3.373 (7)	154
N3—H3···O1	0.88	2.36	3.011 (10)	131

Symmetry codes: (i) *x*−1, *y*, *z*.
